# Exploring the impact of MiR-92a-3p on FOLFOX chemoresistance biomarker genes in colon cancer cell lines

**DOI:** 10.3389/fphar.2024.1376638

**Published:** 2024-04-10

**Authors:** Paula I. Escalante, Luis A. Quiñones, Héctor R. Contreras

**Affiliations:** ^1^ Laboratory of Chemical Carcinogenesis and Pharmacogenetics (CQF), Department of Basic and Clinical Oncology (DOBC), Faculty of Medicine, University of Chile, Santiago, Chile; ^2^ Laboratory of Cellular and Molecular Oncology (LOCYM), Department of Basic and Clinical Oncology (DOBC), Faculty of Medicine, University of Chile, Santiago, Chile; ^3^ Latin American Network for the Implementation and Validation of Pharmacogenomic Clinical Guidelines (RELIVAF), Santiago, Chile; ^4^ Department of Pharmaceutical Sciences and Technology, Faculty of Chemical and Pharmaceutical Sciences, University of Chile, Santiago, Chile; ^5^ Center for Cancer Prevention and Control (CECAN), Santiago, Chile

**Keywords:** miR-92a-3p, DPYD, TYMS, MTHFR, ERCC2, XRCC1, chemoresistance, pharmacoepigenetics

## Abstract

**Introduction:** One of the primary obstacles faced by individuals with advanced colorectal cancer (CRC) is the potential development of acquired chemoresistance as the disease advances. Studies have indicated a direct association between elevated levels of miR-92a-3p and the progression, metastasis, and chemoresistance observed in CRC. We proposed that miR-92a-3p impairs FOLFOX (fluorouracil/oxaliplatin) chemotherapy response by upregulating the expression of chemoresistance biomarker genes through the activation of β-catenin and epithelial-mesenchymal transition (EMT). These FOLFOX biomarker genes include the pyrimidine biosynthesis pathway genes dihydropyrimidine dehydrogenase (*DPYD*), thymidylate synthase (*TYMS*), methylenetetrahydrofolate reductase (*MTHFR*), and the genes encoding the DNA repair complexes subunits *ERCC1* and *ERCC2*, and *XRCC1*.

**Methods:** To assess this, we transfected SW480 and SW620 colon cancer cell lines with miR-92a-3p mimics and then quantified the expression of *DPYD*, *TYMS*, *MTHFR*, *ERCC1*, *ERCC2*, and *XRCC1*, the expression of EMT markers and transcription factors, and activation of β-catenin.

**Results and discussion:** Our results reveal that miR-92a-3p does not affect the expression of *DPYD*, *TYMS*, *MTHFR*, and *ERCC1.* Furthermore, even though miR-92a-3p affects *ERCC2*, *XRCC1*, E-cadherin, and β-catenin mRNA levels, it has no influence on their protein expression.

**Conclusion:** We found that miR-92a-3p does not upregulate the expression of proteins of DNA-repair pathways and other genes involved in FOLFOX chemotherapy resistance.

## 1 Introduction

The FOLFOX chemotherapy scheme is the first-line treatment of metastatic colorectal cancer (CRC), consisting of the association of 5-fluorouracil (5-FU) and oxaliplatin (L-OHP) (Ismaili, 2011; Edwards et al., 2012; Van Cutsem et al., 2016). Unfortunately, for patients diagnosed with distant-stage CRC, 5-year survival is only 14% due to the development of acquired chemoresistance (Hammond et al., 2016; Siegel et al., 2020). A better characterization of the mechanisms involved in acquired chemoresistance could help to prevent and eventually target these mechanisms to improve survival and disease management for distant-stage CRC patients.

It has been reported that activation of the Wnt/β-catenin signaling pathway has the potential to induce resistance to FOLFOX chemotherapy (Hu et al., 2019). As we have reviewed previously, we propose that the Wnt/β-catenin-induced epithelial-mesenchymal transition (EMT) increases the expression of genes directly implicated in the pharmacokinetics and pharmacodynamics of 5-FU and L-OHP (Escalante et al., 2021). These biomarker genes include genes from the pyrimidine biosynthesis pathway dihydropyrimidine dehydrogenase (*DPYD*), thymidylate synthase (*TYMS*), methylenetetrahydrofolate reductase (*MTHFR*), and the genes encoding the DNA repair complexes subunits of the nucleotide-excision repair pathway (NER) *ERCC1* and *ERCC2*, and the complex subunit of the base-excision repair pathway (BER) *XRCC1* (Escalante et al., 2021). The proposed mechanism involves upregulated expression of these biomarker genes by the β-catenin-associated TCF/LEF family of transcription factors and the EMT transcription factors *SNAIL*, *SLUG*, and *ZEB1* (Escalante et al., 2021).

On the other hand, microRNAs have gained interest because of their role in the regulation of gene expression. Among these, miR-92a-3p was found to trigger EMT, inducing activation of β-catenin by downregulating the expression of proteins that co-regulate the β-catenin pathway, including *PTEN* and *FBXW7*, leading to increased migration and invasion in cancer cell lines (Chen et al., 2011; Zhang et al., 2014; Song et al., 2016; Hu et al., 2019). Furthermore, previous studies suggested that miR-92a-3p was linked to metastasis and chemoresistance in CRC patients and animal models (Feng et al., 2018; Hu et al., 2019). According to that, we hypothesized that miR-92a-3p triggers EMT in tumor cells through the activation of β-catenin, leading to the upregulation of *DPYD*, *TYMS*, *MTHFR*, *ERCC1*, *ERCC2*, and *XRCC1* expression.

In the present study, we assessed this hypothesis by transfecting miR-92a-3p mimic and inhibitor oligonucleotides into SW480 (primary tumor) and SW620 (metastatic) colon cancer cell lines. Then, we quantified the expression of FOLFOX biomarker genes *DPYD*, *TYMS*, *MTHFR*, *ERCC1*, *ERCC2*, and *XRCC1* in transfected cells, to determine if miR-92a-3p modulates the expression of these genes as a mechanism of acquired resistance to FOLFOX treatment. In parallel, we determined the expression of the epithelial marker E-cadherin, the EMT transcription factors *SNAIL*, *SLUG*, and *ZEB1*, the expression of total and active β-catenin (ABC), and the mesenchymal marker vimentin to assess EMT and β-catenin activation in response to different levels of miR-92a-3p as a mechanism of regulation of FOLFOX biomarker genes.

## 2 Materials and methods

### 2.1 Cell cultures and transfection experiments

We worked with SW480 (ATCC^®^ CCL-228TM) and SW620 (ATCC^®^ CCL-227TM) colon cancer cell lines. Cells lines were maintained at 37 °C in Gibco™ Leibovitz’s L-15 culture medium supplemented with 10% fetal bovine serum (FBS) in an atmosphere without CO_2_, according to the instructions from the manufacturer.

We seeded 5 × 10^4^ cells per well in 12 well plates for RNA and protein extraction or 2 × 10^4^ cells per well in 24 well plates for immunofluorescence assays until 60% confluence. Cell lines were then transfected with mirVana™ miR-92a-3p-mimic (Cat. # 4464066, Assay Id. MC10916) or mirVana™ mimic negative control oligonucleotide (Cat. # 4464058) at consistent concentrations (20 nM for SW480 cells, 10 nM for SW620 cells) using Lipofectamine 2000™ (Thermo Fisher Scientific) at 1 μL/mL. Additionally, cell lines were transfected independently with mirVana™ miR-92a-3p-inhibitor (Cat. # 4464084, Assay Id. MH10916) or mirVana™ inhibitor negative control oligonucleotide (Cat. # 4464076) at equivalent concentrations (100 nM for both SW480 and SW620 cells) using Lipofectamine 2000™ at 2 μL/mL.

The cells were maintained at 37°C with 5% CO_2_ in Gibco™ Opti-MEM™ reduced serum medium without phenol red during transfection as per the manufacturer’s instructions. The transfection efficiency of miR-92a-3p mimic in SW620 cells was evaluated at 24 and 48 h post-transfection, revealing a significant decrease in miR-92a-3p levels in the cell lines 48 h after transfection (results not presented). Subsequently, transfected cells were either lysed for total RNA and protein extraction or fixed for immunofluorescence experiments.

### 2.2 Reverse transcription and real-time qPCR for mRNA analysis

Total RNA was extracted from transfected SW480 and SW620 cells using RNAzol^®^ RT (Sigma Aldrich, R4533) following the manufacturer’s protocol. Isolated RNA was resuspended in nuclease-free water and quantified using a BioTeK Synergy HT MultiDetection Microplate Reader™ (BioTek, USA). Following that step, RNA reverse transcription was performed in an AriaMx Real-time PCR System (Agilent Technologies, Santa Clara, CA, USA) using the 5x All-In-One RT MasterMix kit (Applied Biological Materials, Bellingham, WA, USA), and product cDNA was diluted into a final concentration of 20 ng/μL.

Afterward, 40 ng of cDNA was used for the analysis of target genes by real-time qPCR in an AriaMx Real-time PCR System (Agilent Technologies, Santa Clara, CA, USA) using the Brilliant II SYBR Green qPCR Master Mix (Agilent Technologies, Santa Clara, CA, USA) following the standard manufacturer’s protocol. Amplification curves for each qPCR reaction were analyzed using the Agilent AriaMx Software 1.71, and the specificity of PCR products was controlled by analyzing the respective melting curves and temperatures. Relative expression of target genes was calculated by normalizing against the housekeeping gene pumilio RNA-binding family member 1 (*PUM1*) using the ΔΔCt method. Primer pair sequences are listed in Supplementary Table S1.

### 2.3 Reverse transcription and real-time qPCR for microRNA analysis

Total RNA was used for reverse transcription in an AriaMx Real-time PCR System (Agilent Technologies, Santa Clara, CA, USA) using the TaqMan™ (Applied Biosystems; Life Technologies, Carlsbad, CA, USA) MicroRNA Reverse Transcription Kit (Cat. # 4366596), TaqMan™ MicroRNA Assay hsa-miR-92a-3p RT-primers (Cat. # 4427975 Assay Id.000431) for reverse transcription of miR-92a-3p, and TaqMan™ MicroRNA Assay U6 RT-primers (Cat. # 4427975 Assay Id: 001973) for reverse transcription of the normalizer U6 RNA. Following this, 10 ng of cDNA was amplified by real-time qPCR in an AriaMx Real-time PCR System (Agilent Technologies, Santa Clara, CA, USA) using the TaqMan™ Universal Master Mix II kit (Cat. # 4440043) and corresponding miR-92a-3p or U6 qPCR primers and probes following the standard protocol. The expression of miR-92a-3p was calculated by normalizing against small nuclear RNA U6 using the ΔΔCt method.

### 2.4 Protein extraction and Western blot

Whole-cell protein was extracted from transfected cells using RIPA lysis buffer supplemented with a protease inhibitor (Roche, Indianapolis, IN, USA). Extracted proteins were quantified using the Bradford protein assay. Denatured proteins (25 µg/lane) were loaded and separated in 10% SDS-PAGE gels and then transferred to a nitrocellulose membrane. Membranes were subsequently blocked in 5% non-fat milk or 5% BSA for 1 h at room temperature and then incubated with primary antibodies overnight at 4°C in a blocking buffer. Bound primary antibodies were detected with secondary antibodies conjugated with horseradish peroxidase (HRP). The protein bands were visualized using an enhanced chemiluminescence detection kit for HRP (EZ-ECL, Biological Industries, Cromwell, CT USA) in a Fusion Fx Imaging System (Vilber Lourmat, Germany). Antibodies used in this work are listed in Supplementary Table S2.

### 2.5 Indirect immunofluorescence

SW480 and SW620 cells (2 × 10^4^) were seeded on 12 mm coverslips until 60% confluence and then transfected as described previously. Non-transfected cells were used as an additional control (only kept in Opti-MEM™ medium during transfection). After transfection, cells were fixed in 4% paraformaldehyde for 30 min. Fixed cells were then permeabilized with 0.1% Triton X-100 in PBS for 10 min, washed, blocked with 3% BSA in PBS for 30 min, and incubated for 15 h with β-catenin antibody (1:100, BD transduction 610154 Mouse antibody). Cells were afterward washed in PBS and incubated with Alexa Fluor 488 secondary antibody (Goat anti-Mouse IgG. 1:200, Cat.#. A21207, Life Technologies) for 1 h. The coverslips were counterstained with 4′,6-diamidino-2-phenylindole (DAPI) and imaged under a confocal microscope C2+ (Nikon, Japan). Images were processed and analyzed using the software ImageJ Ver.2.3.0/1.53q (NIH, Bethesda, MD, USA.). Colocalization images were obtained using the Colocalization Threshold tool in ImageJ.

### 2.6 MTT assay

We seeded 5 × 10³ cells per well in 96 well plates for MTT assays. Cells were cultured until ∼60% confluence and then transfected with miR-92a-3p mimics, inhibitors, and control oligonucleotides, according to the protocol previously defined. Non-transfected cells were used as an additional control for this assay. After 6 h of transfection, 100 μL of Opti-MEM™ culture medium with different concentrations of 5-FU/L-OHP was added to each well until reaching the final concentrations of 0 μM, 25/5 μM, 50/10 μM, 75/15 μM, 100/20 μM, 150/30 μM, 200/40 μM, and 250/50 μM per well (total volume = 200 μL). Treated cells were kept at 37 °C with 5% CO₂ for 24 h. Following that time, we discarded the FOLFOX-Opti-MEM™ culture medium and added 100 μL per well of a solution of MTT 150 μg/mL (Sigma-Aldrich, St Louis, MO, 23 USA) in Locke’s buffer and incubated for 4 h. After this, we discarded the MTT-Locke’s solution and solubilized the resulting formazan crystals in 100 μL DMSO. Absorbancy of the resulting solution was measured at 570 nM using a BioTeK Synergy HT MultiDetection Microplate Reader™ (BioTek, USA).

### 2.7 Statistical analysis

Results are presented as mean ± SEM. Statistical analyses of mRNA and protein expression were performed in the Python programming language Ver. 3.11.5, using the SciPy library Ver. 1.11.2 and the Statsmodels module Vers. 0.14.0. Graphics were created using GraphPad 8.0. Data from viability MTT assays was analyzed with GraphPad 8.0 software.

Gaussian distribution of the data was assessed using Q-Q Plots and the Shapiro-Wilk test, and homoscedasticity using Levene’s test. mRNA and protein relative expression between two data groups was compared using the Student’s t-test or the Kruskal–Wallis H-Test, depending on the results of Gaussian Distribution of the Shapiro-Wilk tests. To assess differences in mRNA and protein relative expression for all four transfection groups we used ANOVA for data with normal distribution, and the Kruskal–Wallis H-Test for non-normal data.

A *p*-value <0.05 was considered significant. All presented data is representative of three independent experiments (n = 3) to ensure that the obtained results are reproducible.

## 3 Results

### 3.1 MiR-92a-3p downregulates the expression of epithelial markers mRNAs but does not induce activation of β-catenin

In order to assess whether miR-92a-3p upregulates the expression of FOLFOX biomarker genes through the β-catenin signaling pathway, we needed to confirm that miR-92a-3p triggers β-catenin activation and EMT. For this purpose, we transiently modified the intracellular levels of miR-92a-3p by transfecting SW480 and SW620 cells with miR-92a-3p mimics, or with miR-92a-3p inhibitors (100 nM = SW480 and SW620).

**FIGURE 1:**
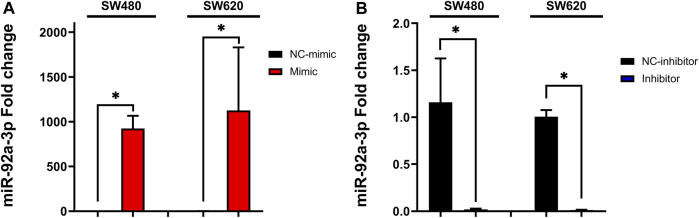
Transfection of SW480 and SW620 cell lines with miR-92a-3p mimic and inhibitor oligonucleotides: SW480 and SW620 cell lines were transfected with mirVana™ (Thermo Fisher Scientific) miR-92a-3p-mimic (Mimic), mimic negative control oligonucleotide (NC-mimic), miR-92a-3p-inhibitor (Inhibitor), inhibitor negative control (NC-inhibitor). Total RNA was extracted 24 h after transfection for quantitative analysis. **(A)** RT-qPCR analysis showing the relative quantity of miR-92a-3p in SW480 and SW620 cells transfected with mimic or negative control. **(B)** RT-qPCR analysis showing the relative quantity of miR-92a-3p in SW480 and SW620 cells transfected with miR-92a-3p inhibitor or negative control. Statistical differences in microRNA levels were determined using the Kruskal–Wallis H-test (*p < 0.05). Data represent the means ± SEM from n = 3 independent experiments.

Relative levels of miR-92a-3p were significantly increased for SW480 (Figure 1A, *p*-value mimic vs. NC-mimic = 0.04953, H-Test), and SW620 (Figure 1A, *p*-value mimic vs. NC-mimic = 0.04953, H-Test) cell lines transfected with miR-92a-3p mimic and significantly decreased for cell lines transfected with miR-92a-3p inhibitors (Figure 1B, *p*-value inhibitor vs. NC-inhibitor = 0.04953, H-Test; SW620 *p*-value inhibitor vs. NC-inhibitor = 0.04953, H-Test). Comparison of means between the four transfection conditions was also assessed by a non-parametric Kruskal–Wallis H-test, confirming that the difference of levels of miR-92a-3p between transfection conditions was statistically significant (SW480 *p*-value = 0.02374, SW620 *p*-value = 0.02374).

**FIGURE 2:**
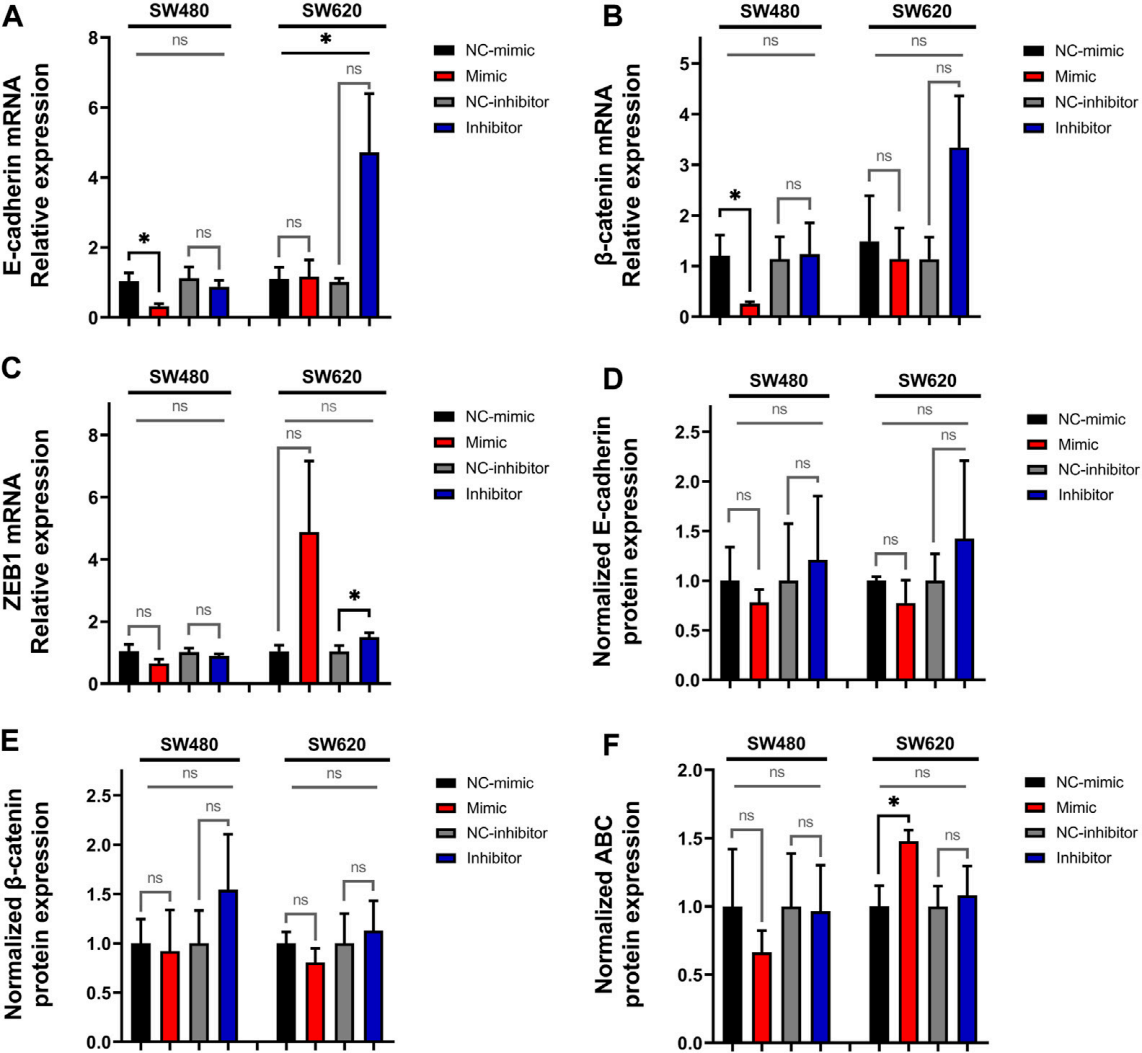
Expression of E-cadherin, β-catenin, and *ZEB1* in SW480 and SW620 cell lines transfected with miR-92a-3p-mimic or inhibitors at mRNA and protein level: SW480 and SW620 cell lines were transfected with mirVana™ (Thermo Fisher Scientific) miR-92a-3p-mimic (Mimic), mimic negative control oligonucleotide (NC-mimic), miR-92a-3p-inhibitor (Inhibitor), inhibitor negative control (NC-inhibitor). Total RNA and proteins were extracted 24 h after transfection for quantitative analysis. RT-qPCR analysis showing the relative mRNA expression of **(A)** E-cadherin, **(B)** β-catenin and **(C)** *ZEB1* upon miR-92a-3p mimic or inhibitor transfection in SW480 and SW620 cells. Western blot analysis showing the relative protein expression of **(D)** E-cadherin, **(E)** β-catenin, and **(F)** ABC upon miR-92a-3p mimic or inhibitor transfection in SW480 and SW620 cells. Statistical differences in mRNA and protein levels were determined using Student’s t-test or Kruskal–Wallis H test for two-group comparisons (square brackets), and ANOVA or Kruskal–Wallis H test for multiple group comparisons (straight superior lines) when corresponding (*p < 0.05). Data represent the means ± SEM from n = 3 independent experiments. ABC: Active β-catenin.

Following this step, we quantified the relative expression of the epithelial marker E-cadherin, the mesenchymal marker vimentin, the EMT transcription factors *SNAIL*, *SLUG* and *ZEB1*, and β-catenin. The results show that transfection with miR-92a-3p mimics downregulates the relative expression of E-cadherin in SW480 cells (Figure 2A, *p*-value mimic vs. NC-mimic = 0.04176, *t*-Test), although these differences were not significant when mimic, inhibitor, and control transfections were compared together by one-way ANOVA (*p*-value = 0.12129). Comparison of the four transfection groups in the SW620 cell line for E-cadherin mRNA shows a significant difference (Figure 2A, ANOVA *p*-value = 0.04895), but not when mimics and inhibitors were individually compared with their respective controls.

Transfection with miR-92a-3p mimics also downregulates β-catenin mRNA in SW480 cells (Figure 2B, *p*-value mimic vs. NC-mimic = 0.04953, H-Test), although these differences were not significant when the four transfection conditions were compared by Kruskal–Wallis H-Test (*p*-value = 0.09433). Transfection with miR-92a-3p inhibitors upregulates the expression of *ZEB1* mRNA in SW620 cells (Figure 2C, *p*-value inhibitor vs. NC-inhibitor = 0.04953, H-Test), although these differences were non-significant when contrasted between mimic, inhibitor, and control transfections (*p*-value = 0.39999, H-Test). The expression of vimentin, *SNAIL*, and *SLUG* mRNA was not modified by transfection of miR-92a-3p mimics and inhibitors (Supplementary Figure S1, *p*-value >0.05 for all experiments).

To confirm the results of the real-time qPCR analysis, we performed Western blot analysis to determine the protein expression of E-cadherin (Figure 2D, Supplementary Figure S2), β-catenin (Figure 2E, Supplementary Figure S2), and ABC (Figure 2F, Supplementary Figure S2). We observed that miR-92a-3p fails to significantly modify the protein expression of E-cadherin and β-catenin in transfected cells (*p*-value >0.05 for all experiments). A statistically significant increase of ABC was observed in SW620 cells transfected with miR-92a-3p mimic (Figure 2 *p*-value mimic vs. NC-mimic = 0.04965, *t*-Test) but was not confirmed by comparison of results of multiple transfection conditions (ANOVA *p*-value = 0.17438).

To further assess whether miR-92a-3p induces EMT through activation of β-catenin, we performed an immunofluorescence assay to detect the subcellular localization of β-catenin in SW480 and SW620 cells transfected with miR-92a-3p mimic or inhibitor, and in untreated (non-transfected) cells.

**FIGURE 3:**
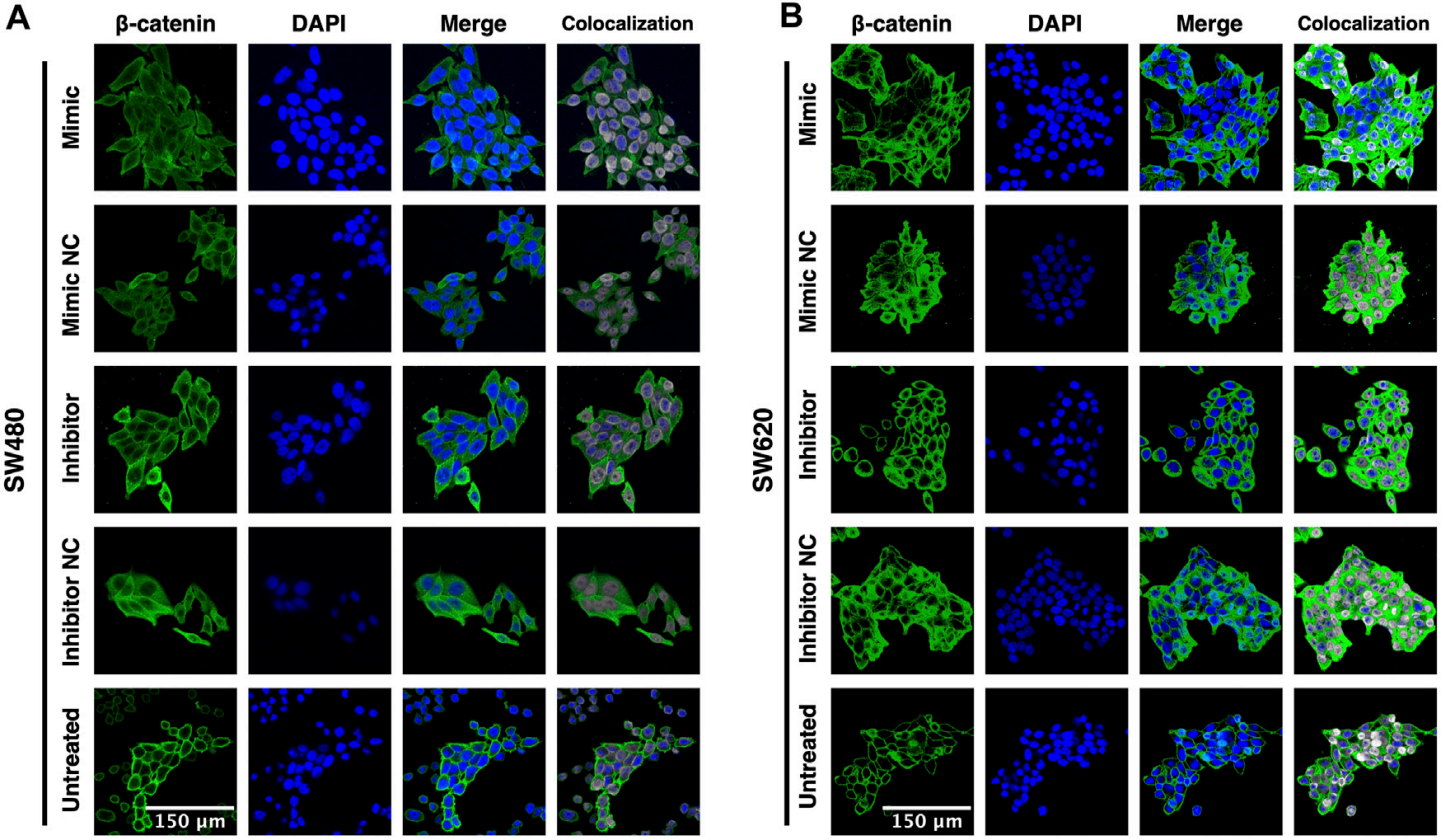
β-catenin subcellular localization in response to miR-92a-3p in SW480 and SW620 cells **(A)** SW480 and **(B)** SW620 cells were transfected with miR-92a-3p-mimic (Mimic), mimic negative control oligonucleotide (NC-mimic), miR-92a-3p-inhibitor (Inhibitor), inhibitor negative control (NC-inhibitor) or kept untreated (Untreated) in Opti-MEM™ culture medium. Transfected and untreated cells were fixed 24 h after for immunofluorescence assay. Green fluorescence corresponds to β-catenin, and DAPI (blue) corresponds to the nuclei as reference. Colocalization images were constructed using the Colocalization Threshold tool, indicating areas of green and blue fluorescence signal overlapping (grey).

The immunofluorescence results show that both, untreated SW480 (Figure 3A) and SW620 (Figure 3B) cell lines display membrane localization of β-catenin, low cytosolic green fluorescence, and nuclear localization of β-catenin in a small proportion of cells. In contrast, all transfected SW480 and SW620 cells show a remarkable increase in cell size and a change in β-catenin distribution to the cytosol with high colocalization of green and blue fluorescence independently of if the cells were transfected with miR-92a-3p mimic, inhibitor, or their corresponding negative controls. These results suggest that β-catenin distribution is probably affected by the transfection process and not by changes in miR-92a-3p levels. Together, these results show that although miR-92a-3p modifies E-cadherin mRNA expression, it fails to activate β-catenin and downregulate E-cadherin protein expression.

### 3.2 MiR-92a-3p does not upregulate the expression of fluoropyrimidine pathway genes

**FIGURE 4:**
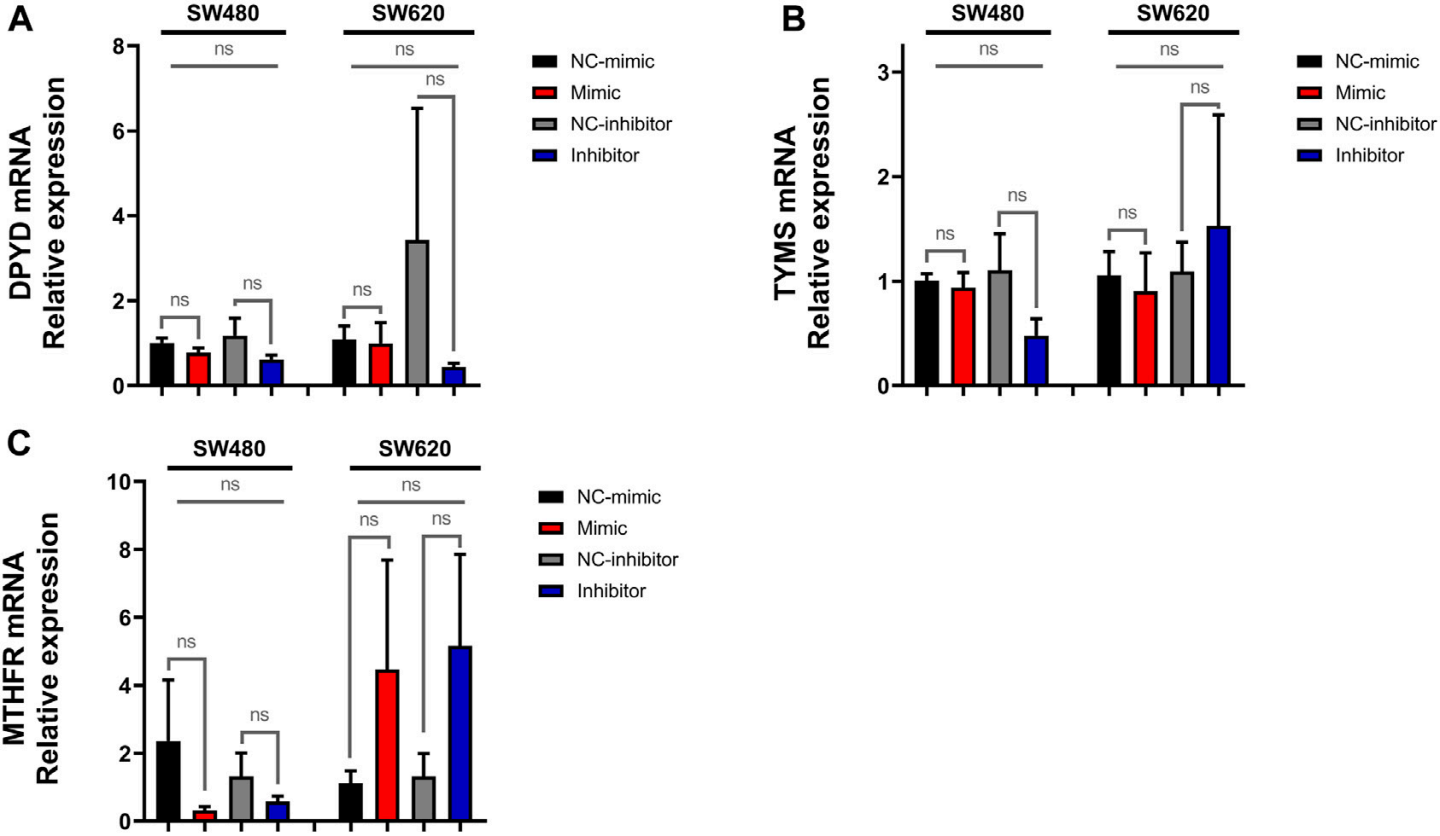
Expression of *DPYD*, *TYMS*, and *MTHFR* in SW480 and SW620 cell lines transfected with miR-92a-3p-mimic or inhibitors: SW480 and SW620 cell lines were transfected with mirVana™ (Thermo Fisher Scientific) miR-92a-3p-mimic (Mimic), mimic negative control oligonucleotide (NC-mimic), miR-92a-3p-inhibitor (Inhibitor), inhibitor negative control (NC-inhibitor). Total RNA and proteins were extracted 24 h after transfection for quantitative analysis. RT-qPCR analysis showing the relative expression of **(A)** *DPYD*, **(B)** *TYMS*, and **(C)** *MTHFR* upon miR-92a-3p mimic or inhibitor transfection in SW480 and SW620 cells. Statistical differences in mRNA and protein levels were determined using Student’s t-test or Kruskal–Wallis H test for two-group comparisons (square brackets) and ANOVA or Kruskal–Wallis H test for multiple group comparisons (straight superior lines) when corresponding (*p < 0.05). Data represent the means ± SEM from n = 3 independent experiments.

To ascertain whether miR-92a-3p affects the expression of genes involved in the pharmacokinetics and pharmacodynamics of 5-FU and L-OHP, we analyzed the mRNA expression of *DPYD* (Figure 4A), *TYMS* (Figure 4B), and *MTHFR* (Figure 4C) in the transfected SW480 and SW620 cells by real-time qPCR, which showed no significant changes in mRNA levels in all experimental conditions (*p*-value >0.05 for all experiments). Together, these results suggest that the mRNA expression of *DPYD*, *TYMS*, and *MTHFR* is not modified by miR*-*92a-3p SW480 and SW620 cells.

### 3.3 MiR-92a-3p downregulates the expression of DNA repair-complex subunits mRNAs

**FIGURE 5:**
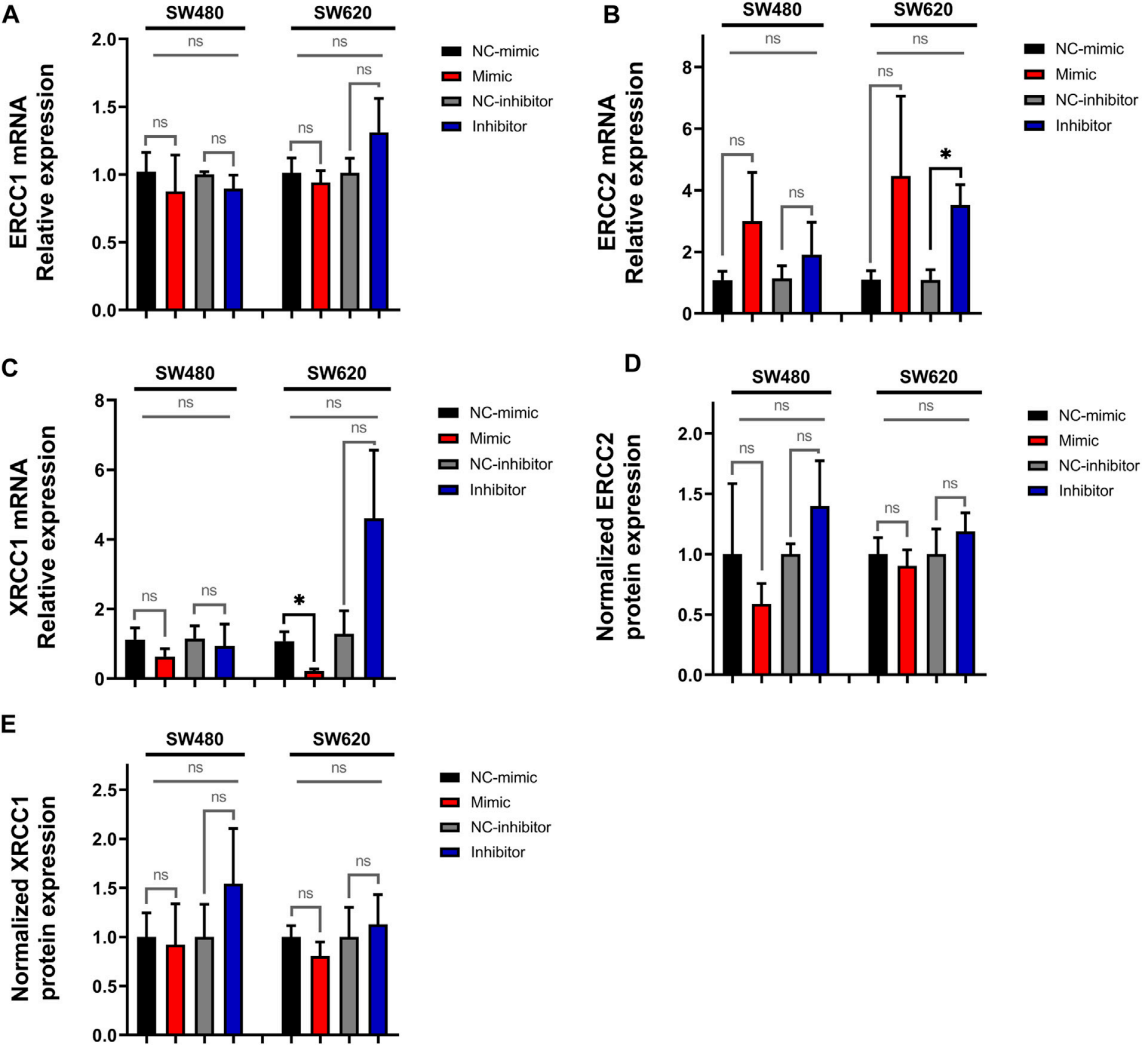
Expression of *ERCC1*, *ERCC2* and *XRCC1* in SW480 and SW620 cell lines transfected with miR-92a-3p-mimic or inhibitors at mRNA and protein level: SW480 and SW620 cell lines were transfected with mirVana™ (Thermo Fisher Scientific) miR-92a-3p-mimic (Mimic), mimic negative control oligonucleotide (NC-mimic), miR-92a-3p-inhibitor (Inhibitor), inhibitor negative control (NC-inhibitor). Total RNA and proteins were extracted 24 h after transfection for quantitative analysis. RT-qPCR analysis showing the relative mRNA expression of **(A)** *ERCC1*, **(B)** *ERCC2,* and **(C)** *XRCC1* upon miR-92a-3p mimic or inhibitor transfection in SW480 and SW620 cells. Western blot analysis showing the relative protein expression of **(D)** *ERCC2* and **(E)** *XRCC1* upon miR-92a-3p mimic or inhibitor transfection in SW480 and SW620 cells. Statistical differences in mRNA and protein levels were determined using Student’s t-test or Kruskal–Wallis H test for two-group comparisons (square brackets) and ANOVA or Kruskal–Wallis H test for multiple group comparisons (straight superior lines) when corresponding (*p < 0.05). Data represent the means ± SEM from n = 3 independent experiments.

We next analyzed the expression of *ERCC1*, *ERCC2*, and *XRCC1* in transfected SW480 and SW620 cells. The results show a significant increase in *ERCC2* mRNA levels in SW620 cells transfected with miR-92a-3p inhibitor (Figure 5B, *p*-value inhibitor vs. NC-inhibitor = 0.03009, *t*-Test), which was not confirmed by analysis of multiple transfection conditions (ANOVA *p*-value = 0.26532). We also observed a significant decrease in *XRCC1* mRNA relative expression in SW620 cells transfected with miR-92a-3p-mimic (Figure 5C, *p*-value mimic vs. NC-mimic = 0.03439, *t*-Test), which again was not confirmed by ANOVA results (*p*-value = 0.07253). Expression of *ERCC1*, on the other hand, was not affected by transfection with miR-92a-3p mimic or inhibitor (Figure 5A).

To confirm these results, we assessed *ERCC2* and *XRCC1* protein expression by Western blot. *ERCC2* (Figure 5D, Supplementary Figure S2) and *XRCC1* (Figure 5E, Supplementary Figure S2) protein levels were not significantly up or downregulated in SW480 and SW620 cells transfected with miR-92a-3p-mimics or inhibitors compared with controls (*p*-value >0.05 for all experiments. Taken together, these results reveal that miR-92a-3p downregulates the expression of the DNA-repair complexes subunits *ERCC2* and *XRCC1* mRNAs but has no effect in *ERCC1* mRNA expression, and it fails to exert an effect on the protein expression of these genes.

## 4 Discussion

Previous research has highlighted that miR-92a-3p has numerous and versatile effects on tumor cells. Our results show that rather than increasing the expression of FOLFOX biomarker genes, which is the expected result according to our proposed hypothesis, there is an inverse correlation between miR-92a-3p and the expression of *ERCC2* and *XRCC1* mRNA only in SW620 cells (Figures 5B, C). The SW620 cell line represents a more advanced disease stage (lymph node metastasis) compared to the SW480 cell line (primary tumor) which suggests that the response to miR-92a-3p is affected by the degree of tumor progression. Nonetheless, these mRNA changes did not lead to changes in the protein levels of these genes.

As a proof of concept for potential changes in the FOLFOX sensibility of SW480 and SW620 cell lines mediated by miR-92a-3p, we performed an MTT assay for all four transfection conditions and contrasted the results with non-transfected SW480 and SW620 cells (Supplementary Figure S3). The results show some significant differences in viability percentage for some isolated 5-FU/L-OHP concentrations (Supplementary Figure S3A, Supplementary Figure S3D). A significant shift in the effective concentration of 5-FU/L-OHP required to decrease viability of each cell line by 50% (EC50) was observed for the SW480 cell line transfected with miR-92a-3p mimic (Supplementary Figure S3E, EC50 ratio = 4.122, 95% CI = 2.516–7.505, *p*-value = 0.0444) but also for SW480 cell line transfected with mimic control oligonucleotide (Supplementary Figure S3E, EC50 ratio = 2.685, 95% CI = 1.867 to 4.266, *p*-value = 0.0026). These results indicate that the EC50 for SW480 cells transfected with miR-92a-3p mimic is increased ∼4 times compared with non-transfected cells, but paradoxically, the EC50 for the same cell line transfected with control oligonucleotides is also increased by ∼2 times, and no statistically significant difference was observed between transfection with miR-92a-3p mimic and control. EC50 shifts for SW480 cells transfected with miR-92a-3p inhibitor or inhibitor control, as well as the EC50 shifts for all transfection conditions on the SW620 cell line showed no significant difference (*p*-value> 0.05).

These results display a notable divergence from previous research, where it is expected to observe decreased viability of cell lines because of the cellular stress and membrane disruption induced by transfection (Jin et al., 2015; Lee et al., 2019). This contradictory phenomenon could be explained by the acquisition of surviving SW480 and SW620 cells of what is known as a senescence-associated secretory phenotype (SASP). This SASP phenotype has been observed in cancer cells exposed to chemo and radiotherapy, displaying proliferation arrest, secretory phenotype, and high metabolic activity, which includes MTT reactant metabolization, and results in treatment sensitivity underestimation (Mirzayans et al., 2017; Ghasemi et al., 2021). Although this phenomenon is considered a limitation of the MTT assay, we hypothesize that transfection may induce enough stress over SW480 and SW620 cells to induce SASP phenotype, which would explain not only the unexpected viability assay results but also the enlarged cells observed in the immunofluorescence experiments, which were not exposed to 5-FU/L-OHP. Further research is required to establish the effect of mimic/inhibitor transfection oligonucleotides on *in vitro* cell behavior.

In later analysis, we quantified the relative protein expression of two mir-92a-3p targets, *KLF4* and *REST*, in protein samples of mimic and inhibitor-transfected SW480 and SW620 cell lines (Supplementary Figure S4). Our results show that although a tendency in the regulation of protein translation of *KLF4* by mir-92a-3p was found (Supplementary Figures S4A, C), the difference in protein expression is still not statistically significant in all transfection conditions, and for *REST* (Supplementary Figures S4B, C) these tendencies are even less prominent. Xiao *et al.* (Jin et al., 2015) reported that *in vitro* transfection of microRNA mimics at low concentrations failed to suppress target gene expression, while transfection at higher concentrations of microRNA mimics altered gene expression in a non-specific manner and led to an accumulation of high-weight RNA species, compared to other strategies of microRNA-expression modulation. The results presented in this research work serve us to further question the functional suitability of *in-vitro* transfection of predesigned microRNA mimic oligonucleotides as an *in-vitro* experimental strategy.

We also explored several *in silico* target predictions databases, including miRDB (https://mirdb.org/), MicroT (https://dianalab.e-ce.uth.gr/microt_webserver/#/interactions), miRPathDB 2.0 (https://mpd.bioinf.uni-sb.de/), and miRTargetLink 2.0 (https://ccb-compute.cs.uni-saarland.de/mirtargetlink2/), to find potential targets for miR-92a-3p between our genes of interest. Most predicted interactions were found in miRPathDB (E-cadherin, *SNAIL*, *SLUG*, *ZEB1*, *DPYD*, and *ERCC1*) and listed as predicted union, except for E-cadherin, which has been experimentally assessed with strong evidence. MiRDB and MicroT showed predicted miRNA response element (MRE) sequences in the 3′-UTR and coding sequences of *SLUG* mRNA, and miRDB showed a predicted MRE sequence in the 3′-UTR sequence of *SNAIL* mRNA. MiRTargetLink shows experimentally assessed regulation of E-cadherin by miR-92a-3p (Chen et al., 2011) and weak evidence of non-canonical interaction with β-catenin (Helwak et al., 2013).

These prediction targets provide no further explanation for the results observed in this work. We did not observe a clear regulation of *SNAIL*, *SLUG*, *DPYD*, and *ERCC1* mRNA in transfected SW480 and SW620 cell lines, which although would have been against our original hypothesis, also would have revealed that these genes are susceptible to miR-92a-3p RISC-dependent regulation. On the other hand, *ERCC2* and *XRCC1* mRNAs, which do not contain a miR-92a-3p MRE in 3′-UTR, appear to be regulated or at least be inversely correlated with miR-92a-3p intracellular levels.

The TFIIH core complex helicase is composed of *ERCC2* (excision repair cross-complementation group 2) together with the xeroderma pigmentosum group B-complementing protein (*XPB*) and is part of the NER DNA-repair pathway (Marsh et al., 2009; Di Francia et al., 2015). On the other hand, *XRCC1* (X-ray repair cross-complementing 1) is a subunit that associates with DNA ligase III (Lig III) as part of the BER pathway (Marsh et al., 2009; Di Francia et al., 2015). Both DNA repair pathways have been previously associated with resistance to oxaliplatin and other platinum-derived drugs (Paré et al., 2008; Gnoni et al., 2011; Di Francia et al., 2015; Hammond et al., 2016; López-Cortés et al., 2020). Per this background and based only on the observed changes of *ERCC2* and *XRCC1* mRNA expression, we may hypothesize that miR-92a-3p improves platinum sensitivity in colon cancer cell lines by downregulation of *ERCC2* and *XRCC1* expression. Yet, this hypothesis contradicts previous research indicating that miR-92a-3p promotes FOLFOX chemoresistance in SW480 and SW620 cell lines (Hu et al., 2019).

Still, *XRCC1* was found to be downregulated in ESCC tissue samples and cell lines with higher expression of mesenchymal markers and phenotype (Zhang et al., 2020), suggesting that DNA repair may be impaired during EMT. Moreover, a recent study explored the downregulation of DNA-repair pathways in a model of acquired resistance to targeted therapies. Under the pressure of prolonged treatment with targeted therapies, such as cetuximab, cancer cells exhibited lower DNA-repair competence by down-modulating the mismatch repair and homologous recombination DNA-repair pathways and expression of low-fidelity DNA polymerases. Together, this led to permanent DNA instability and progressive accumulation of genomic damage in persistent cancer cells, which in return increased the chance of acquiring genetic resistance to these targeted therapies, developing a stress-induced hypermutability phenotype (Russo et al., 2019; Gay et al., 2020).

Through a different mechanism, long-term chemotherapy may induce chemoresistance by upregulation of miR-92a-3p, and subsequent downregulation of *ERCC2* and *XRCC1* expression in colon cancer, promoting hypermutability and tumor heterogeneity. MiR-92a-3p has shown to target sirtuin 1 (*SIRT1*), a class III histone deacetylase, in human endothelial cell lines (Chen et al., 2015). In a separate study, *SIRT1* inhibited proteasomal degradation of *XRCC1*, leading to cisplatin resistance in lung cancer cell lines (Yousafzai et al., 2019). Together, both studies describe a potential interaction between miR-92a-3p and *XRCC1* expression, although these results do not align with our Western blot results (Figure 5E). Decreased DNA repair through a downregulated expression of the NER and BER subunits *ERCC2* and *XRCC1* may promote hypermutability while increasing platinum sensitivity at the same time. The mechanisms by which miR-92a-3p downregulates *ERCC2* and *XRCC1* mRNA expression and whether this may lead to decreased protein expression and NER/BER DNA-repair proficiency have yet to be explored.

In this study, we observed an indirect correlation between levels of miR-92a-3p and expression of E-cadherin mRNA in SW480 and SW620 cell lines that may be evidence of direct targeting of E-cadherin mRNA by miR-92a-3p (Figure 2A). In epithelial cells, E-cadherin and β-catenin are localized in the cell membrane as part of the cell-to-cell interactions known as adherents’ junctions. Downregulated E-cadherin promotes EMT by increasing the amount of free β-catenin in the cell cytosol (Brembeck et al., 2006; Valenta et al., 2012; Gonzalez and Medici, 2014). Yet, our results show that E-cadherin protein is not downregulated, nor total β-catenin and ABC proteins are upregulated in transfected SW480 and SW620 cells.

An alternative mechanism by which miR-92a-3p triggers EMT is through *PTEN* targeting and subsequent activation of the Pi3K/Akt signaling pathway, which has been shown to promote migration and invasiveness in colorectal cancer, glioma, and ESCC cell lines (Zhang et al., 2014; Ke et al., 2015; Li et al., 2019). Conversely, it has also been reported that miR-92a-3p inhibits invasion, migration, and proliferation by targeting *SOX4* in prostate cancer cell lines (Liao et al., 2020) and by targeting Notch1 in glioma stem cells (Song et al., 2016) and Wilms tumor cells (Zhu et al., 2018), showing that despite the pro-tumoral role that has been generally described for miR-92a-3p, the cell type and experimental approach may be more than relevant in defining the response to this microRNA.

A challenge when establishing the impact of a particular microRNA in disease is the pleiotropic effect of microRNA regulation of gene expression in the cell. The miR-17–92 cluster, which encodes miR-92a-3p, is a highly preserved microRNA cluster containing six tandem microRNAs, miR-17, miR-18a, miR-19a, miR-20a, miR-19b-1, and miR-92a, which are co-transcribed as a unique pri-miR. Previous findings indicate that both miR-17-5p and miR-20a-5p initiate a regulatory loop that negatively modulates the expression of the whole tandem, and evidence shows that miR-17-5p also downregulates the prooncogenic effects of miR-92a-3p, which could provide a potential explanation of why and how the response to miR-92a-3p transfection was counteracted (Chaulk et al., 2011; Chakraborty and Krishnan, 2017).

**FIGURE 6:**
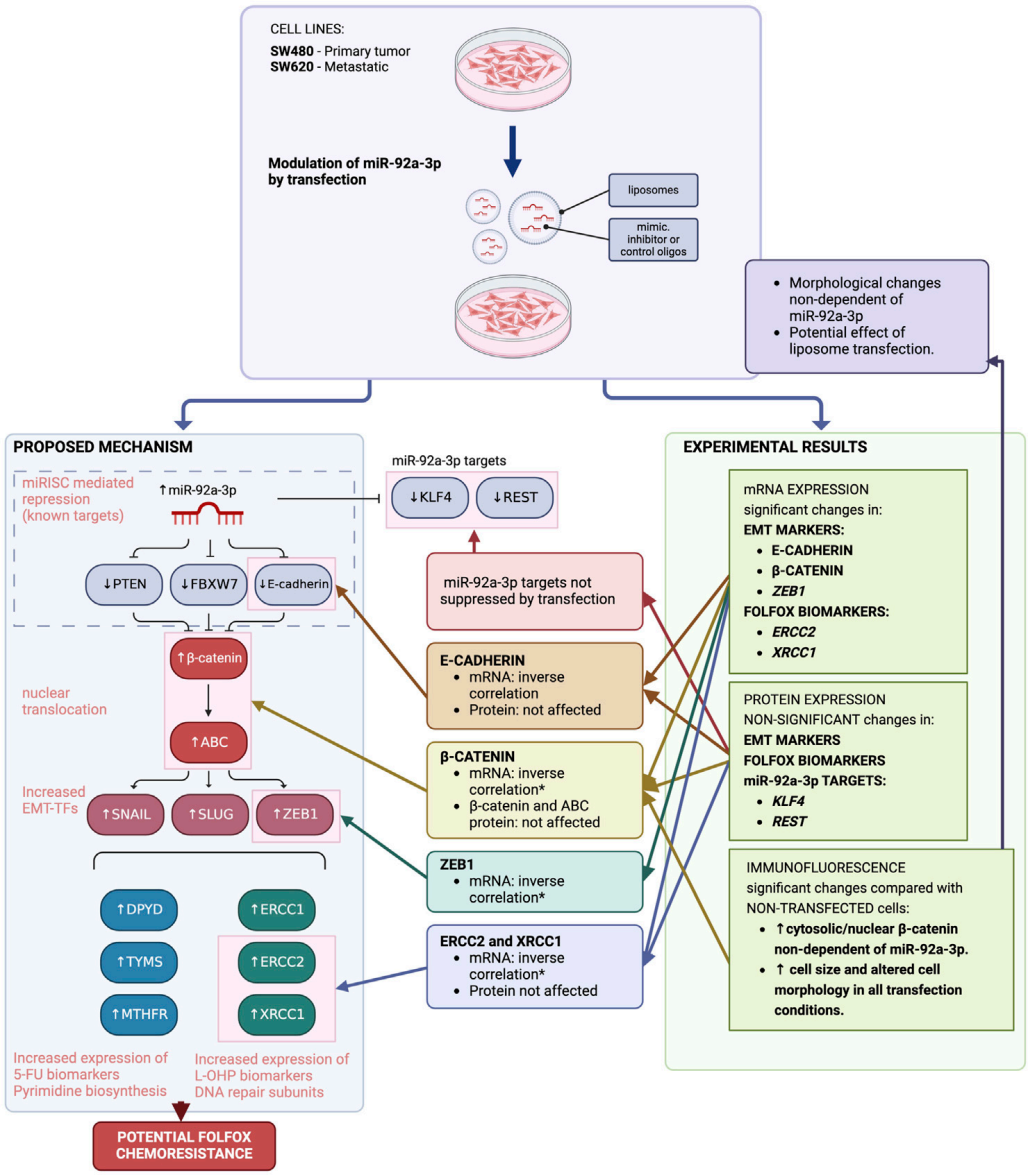
Impact of MiR-92a-3p on FOLFOX chemoresistance biomarker genes in colon cancer cell lines (SW480 and SW620), proposed hypothesis vs. experimental results. The suggested mechanism posits that in colon cancer cell lines, elevated miR-92a-3p levels due to cancer progression inhibit the expression of specific targets - *PTEN*, *FBXW7*, and E-cadherin - through miRISC complex translational suppression. This leads to heightened levels of free cytosolic β-catenin within tumor cells. The active form of β-catenin in the nucleus promotes the expression of certain transition-transcription factors, which subsequently trigger the overexpression of genes associated with resistance to FOLFOX chemotherapy. Experimental findings from transfection studies indicate that while mRNA levels of certain genes show an inverse relationship with miR-92a-3p levels, protein expression remains unaltered. Furthermore, the transfection of miR-92a-3p did not affect the translation of its known targets. Immunofluorescence assays demonstrated changes in cellular morphology and β-catenin distribution, which may be attributed to the impact of transfection procedures on cell integrity, rather than miR-92a-3p modulation. Adapted from Escalante *et al.* (Escalante et al., 2021). Image created with BioRender.com.

The role of miR-92a-3p has been explored for multiple neoplasia, with often contradictory outcomes (Chen et al., 2011; Tsuchida et al., 2011; Zhang et al., 2014; Ke et al., 2015; Song et al., 2016; Fu et al., 2018; Zhu et al., 2018; Hu et al., 2019; Li et al., 2019; Wei et al., 2019; Liao et al., 2020). In CRC, current evidence suggests that this microRNA participates in disease progression, and potentially in acquired chemoresistance (Tsuchida et al., 2011; Fu et al., 2018; Hu et al., 2019). We aimed to assess how miR-92a-3p affects the expression of multiple FOLFOX biomarker genes to ascertain the role of this microRNA in the development of FOLFOX chemoresistance, which is more prevalent in patients diagnosed with metastatic CRC (Hammond et al., 2016; Siegel et al., 2020). Establishing the role of miR-92a-3p and other microRNAs in cancer progression has the potential to unveil a whole spectrum of therapeutic tools, from monitoring the early steps of chemoresistance to novel microRNA-based treatments (Hanna et al., 2019). However, the mechanisms hereby explored did not reveal an association between miR-92a-3p and upregulated expression of FOLFOX biomarkers. If anything, the results of our investigation bring to light a significant issue associated with transfection experimental strategies. These results, summarized in Figure 6, demonstrate the potential problems that can arise from these strategies when evaluating a particular type of hypothesis. Moreover, they emphasize the significance of comparing the obtained results with appropriate experimental controls.

Certain limitations of our study need to be addressed. Specifically, the sample size in some experiments may be too limited to draw definitive conclusions. A statistical power analysis was conducted to assess this issue, confirming that the selected sample size is indeed restricted. This constraint may account for the absence of significant trends in mRNA expression levels of *ERCC2*, *SLUG*, and vimentin in SW620 and SW480 cells transfected with the miR-92a-3p mimic. It is evident that both the sample size and experimental methodology placed constraints on the evaluation of hypotheses in these experiments. However, expanding the sample size at this juncture is currently unfeasible.

These findings also highlight a potential issue with the reproducibility of previous research results, given our anticipations of observing miR-92a-3p-mediated β-catenin activation in our transfection outcomes (Song et al., 2016; Hu et al., 2019). The brief transfection duration likely plays a significant role in the lack of significant β-catenin activation observed in our experiments. Overall, these observations underscore some of the limitations inherent in transfection as an experimental technique, especially when evaluating alterations in protein expression levels.

Because of the limitations of transfection when using microRNA mimics and inhibitors, a different experimental strategy, like designed plasmids or viral vectors for expression of miR-92a-3p, could potentially give more time and be functionally more effective for the microRNA to exert measurable changes in protein expression. This limitation is also relevant when assessing the results of the immunofluorescence assays, where the shifts in β-catenin subcellular distribution may be a consequence of changes in cellular behavior or by direct disruption of the cell membrane integrity by incorporation of liposomes during the transfection process, which could explain the enlarged cell size of transfected cells (Lv et al., 2006; Lee et al., 2019). Using plasmids or viral vectors rather than transient transfection may give the cells enough time to recover their regular cell membrane structure before analyzing the response to changes in miR-92a-3p levels.

In summary, in our approach no effect of miR-92a-3p was observed on *DPYD*, *TYMS*, and *MTHFR* expression. The present study showed an inverse correlation between miR-92a-3p and *ERCC2* and *XRCC1* mRNA expression in SW620 colon cancer cells, which itself proposes a gap in knowledge about the mechanisms of acquired chemoresistance to long-term FOLFOX chemotherapy. However, these changes were not enough to cause changes in the protein expression of these genes. Improvements in the experimental design may be necessary to assess the role of miR-92a-3p in the emergence of chemoresistance in advanced CRC patients.

## Data Availability

The datasets presented in this study can be found in online repositories. The names of the repository/repositories and accession number(s) can be found below: https://github.com/PaulaEscalante/Python-statistical-data-analysis.
