# Supplementary material for: Exploring the impact of MiR-92a-3p on FOLFOX chemoresistance biomarker genes in colon cancer cell lines
Source: Front Pharmacol. 2024 Apr 10;15:1376638. doi: 10.3389/fphar.2024.1376638 (PMC11039864; doi:10.3389/fphar.2024.1376638)
Supplement: Supplementary file 1 [file DataSheet1.docx]

Exploring the Impact of MiR-92a-3p on FOLFOX Chemoresistance Biomarker Genes in Colon Cancer Cell Lines.

Paula I. Escalante ^1,2^, Luis A. Quiñones ^1,3,4^,* and Héctor R. Contreras ^2,5*^

^1^Laboratory of Chemical Carcinogenesis and Pharmacogenetics (CQF), Department of Basic and Clinical Oncology (DOBC), Faculty of Medicine, University of Chile, 8500000, Santiago, Chile.

^2^Laboratory of Cellular and Molecular Oncology (LOCYM), Department of Basic and Clinical Oncology (DOBC), Faculty of Medicine, University of Chile, 8380453, Santiago, Chile.

^3^Latin American Network for the Implementation and Validation of Pharmacogenomic Clinical Guidelines (RELIVAF-CYTED), 28015, Madrid, Spain.

^4^ Department of Pharmaceutical Sciences and Technology, Faculty of Chemical and Pharmaceutical Sciences, University of Chile, Santiago, Chile

^5^ Center for Cancer Prevention and Control (CECAN). Santiago, Chile 8380453

*** Correspondence:**Luis A. Quiñones
lquinone@uchile.cl

Héctor R. Contreras
hcontrer@uchile.cl

**Supplementary Table 1:** List of primer sequences used for real-time qPCR analysis.

| **Gene** | **Forward primer (5’-3’)** | **Reverse primer (5’-3’)** |
| --- | --- | --- |
| *DPYD* | CCG AGA AGC AAT GAG ATG CCT | ACA CAA AGA TCA GAG GTT GGA CA |
| *TYMS* | TGG GGC AGA TCC AAC ACA TC | TTT GTG GAT CCC TTG ATA AAC CAC |
| *MTHFR* | GGC CAT CTG CAC AAA GCT AAG | AAC TCA CTT CGG ATG TGC TTC AC |
| *ERCC1* | TTG GCG ACG TAA TTC CCG ACT AT | TTC ACA TCC ACC TGG ACA AGC AG |
| *ERCC2* | CTT GCT CGA TAC TCA ATC CTG C | ATG GAG TCG ATG CAG ACG TT |
| *XRCC1* | GGC AGA CAC TTA CCG AAA ATG G | GGA CAT GAA AGA TGA GGT GAC CA |
| *SNAIL* | GAG CTG CAG GAC TCT AAT CCA GAG | AGC CTG GAG ATC CTT GGC CTC AG |
| *SLUG* | CAA GGA ATA TGT GAG CCT GGG CG | TCA GTG TGC TAC ACA GCA GCC A |
| *ZEB1* | GAT GAT GAA TGC GAG TCA GAT GC | ACA GCA GTG TCT TGT TGT TGT |
| E-cadherin | TCC CAA CTC CTC TCC TGG CCT | GAG GCT CTG TCA CCT TCA GCC A |
| β-catenin | CGA GCT GCT ATG TTC CCT GA | TCA GCC AAA CGC TGG ACA TT |
| Vimentin | TGG ACC AGC TAA CCA ACG AC | GCC AGA GAC GCA TTG TCA AC |
| Pumilio | CGG TCG TCC TGA GGA TAA AA | CGT ACG TGA GGC GTG AGT AA |

**Supplementary Table 2:** Antibodies used for western blot.

| **Antibody** | **Brand** | **Catalog #** | **Dilution** | |
| --- | --- | --- | --- | --- |
| *PRIMARY ANTIBODIES* | | | | |
| ERCC2 | Cell Signalling | 11963S | 1:2000 | |
| XRCC1 | Cell Signalling | 2735S | 1:500 | |
| E-cadherin | BD Transduction Laboratories | 610181 | 1:1000 | |
| β-catenin | BD Transduction Laboratories | 610154 | 1:1000 | |
| Active β-catenin | Millipore | 05-665 | 1:1000 | |
| KLF4 | Abcam | ab215036 | 1:1000 | |
| REST | Abcam | ab21635 | 1:1000 | |
| β-actin | MP Biomedicals | 691002 | 1:10.000 | |
| *SECONDARY ANTIBODIES* | | | | |
| Goat anti-Mouse IgG HRP | Jackson Immunoresearch | 115-0335-003 | 1:10.000 | |
| Goat anti-Rabbit IgG HRP | Jackson Immunoresearch | 111-035-003 | 1:10.000 | |

**
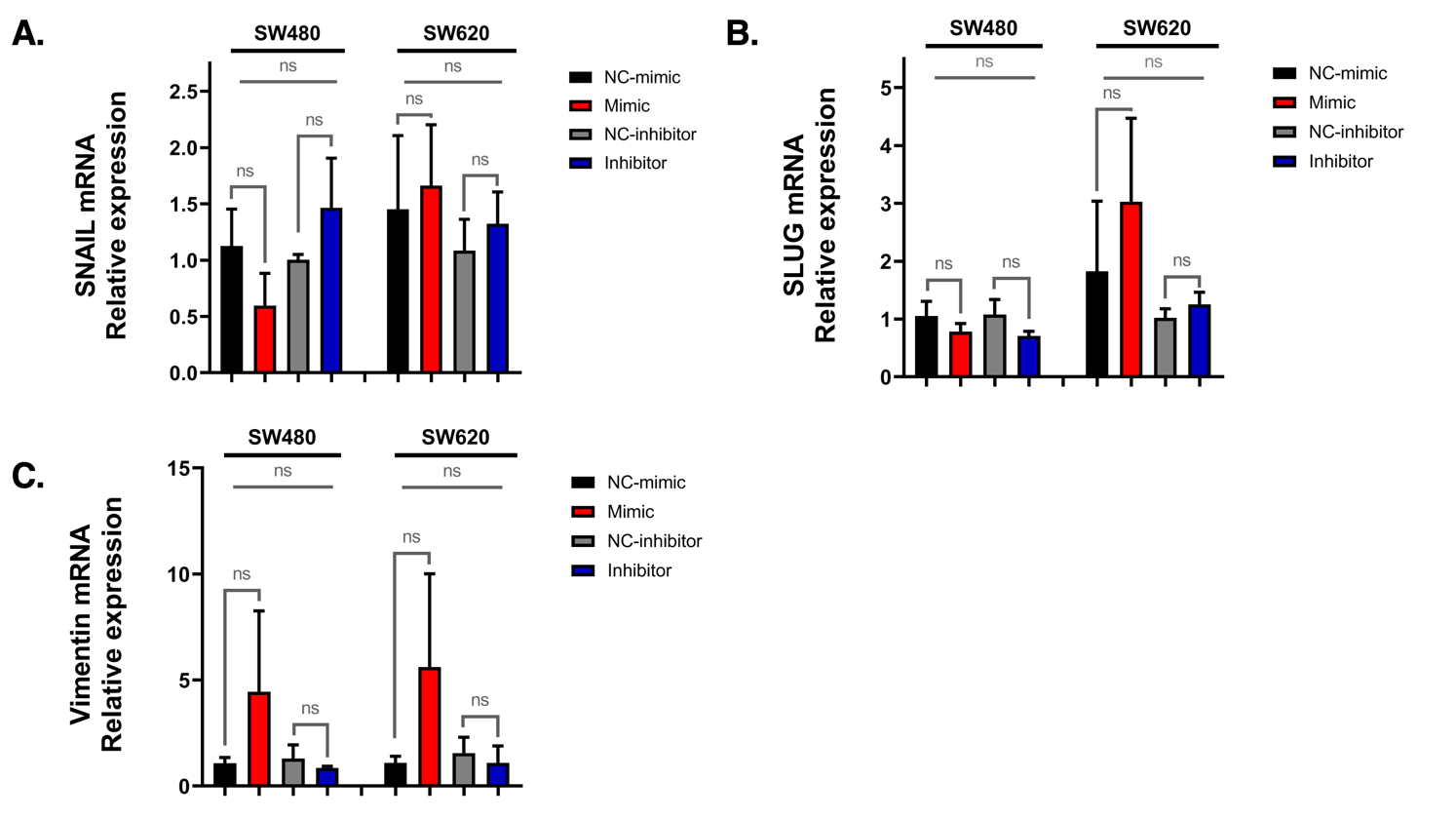
**

**Supplementary Figure S1. Expression of *SNAIL*, *SLUG*, and Vimentin in SW480 and SW620 cell lines transfected with miR-92a-3p-mimic or inhibitors:** SW480 and SW620 cell lines were transfected with mirVana™ (Thermo Fisher Scientific) miR-92a-3p-mimic (Mimic), mimic negative control oligonucleotide (NC-mimic), miR-92a-3p-inhibitor (Inhibitor), inhibitor negative control (NC-inhibitor). Total RNA was extracted 24 hours after transfection for quantitative analysis. RT-qPCR analysis showing the relative expression of (A) *SNAIL*, (B) *SLUG*, and (C) Vimentin upon miR-92a-3p mimic or inhibitor transfection in SW480 and SW620 cells. Statistical differences in mRNA and protein levels were determined using Student’s T-Test or Kruskal-Wallis H test for two-group comparisons (square brackets) and ANOVA or Kruskal-Wallis H test for multiple group comparisons (straight superior lines) when corresponding (*p<0.05). Data represent the means ± SEM from n=3 independent experiments.

**
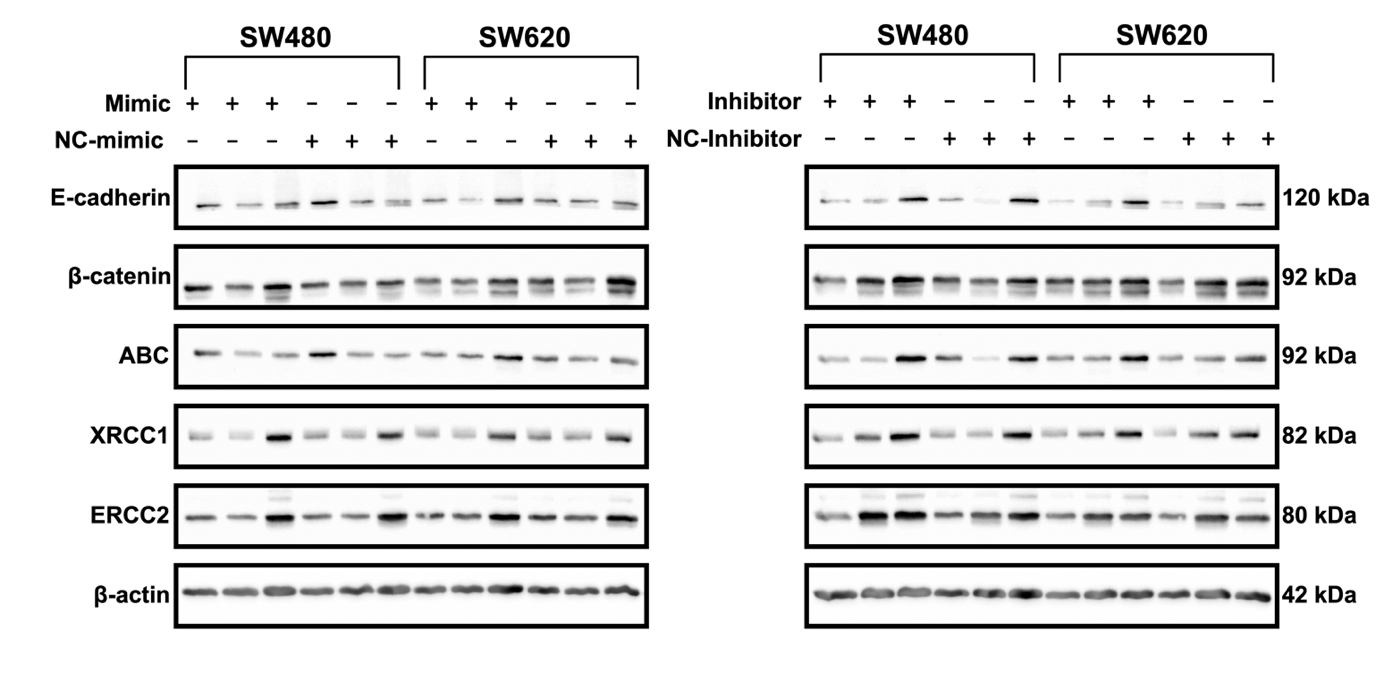
**

**Supplementary Figure S2. Protein quantification of ABC, E-cadherin, β-catenin, *ERCC2* and *XRCC1* in SW480 and SW620 cell lines transfected with miR-92a-3p-mimic or inhibitors:** SW480 and SW620 cell lines were transfected with mirVana™ (Thermo Fisher Scientific) miR-92a-3p-mimic (Mimic), mimic negative control oligonucleotide (NC-mimic), miR-92a-3p-inhibitor (Inhibitor), inhibitor negative control (NC-inhibitor). Total proteins were extracted 24 hours after transfection for quantitative analysis. Western blotting of E-cadherin, β-catenin, ABC, *ERCC2*, *XRCC1*, and β-actin from three independent experiments of SW480 and SW620 cells transfected with miR-92a-3p-mimic (A) or miR-92a-3p-inhibitor (B) and contrasted with their respective control oligonucleotides. (ABC= active β-catenin)

**
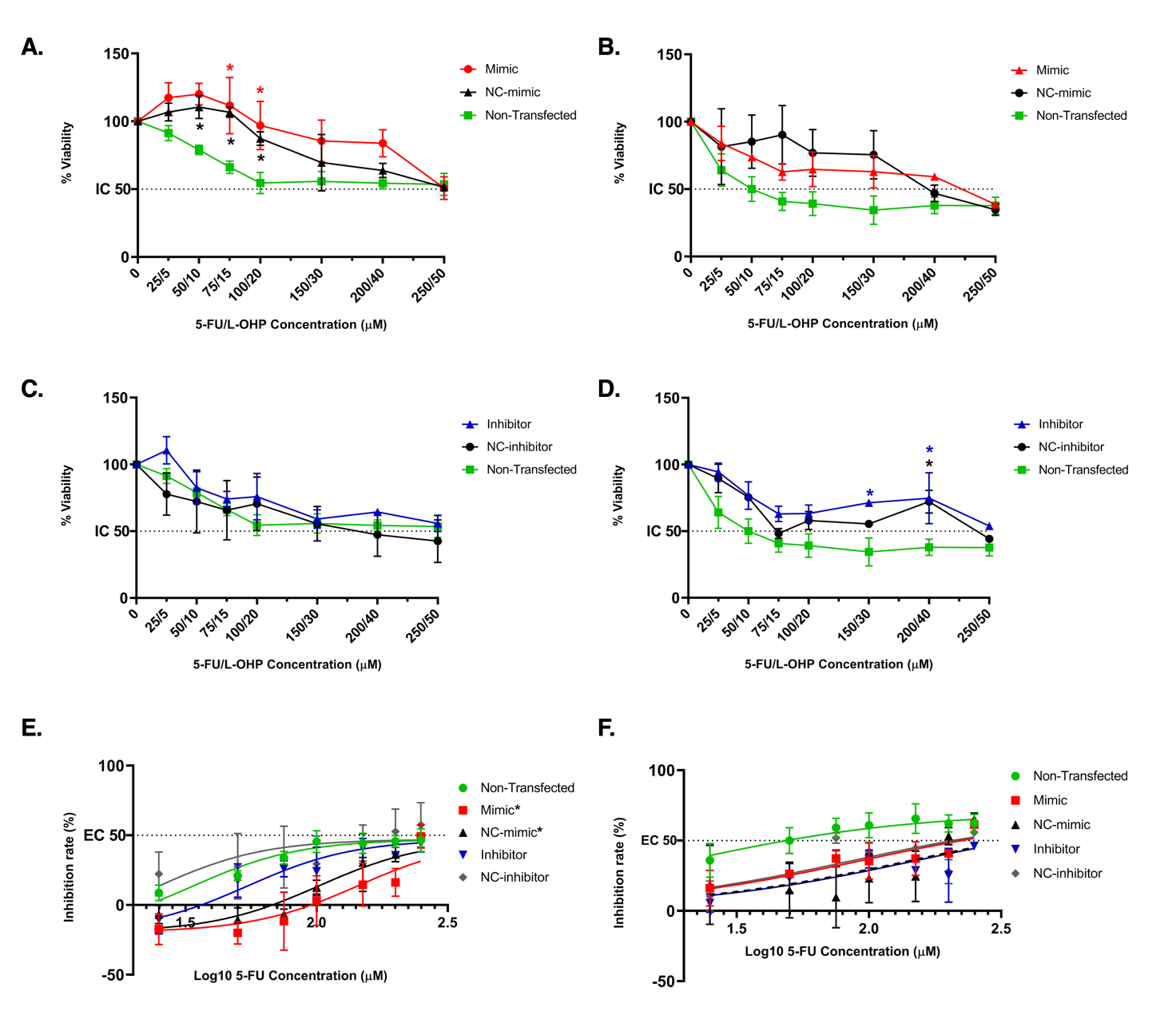
**

**Supplementary Figure S3. Viability of SW480 and SW620 cell lines transfected with miR-92a-3p-mimic or inhibitors to *in-vitro* 5-FU/L-OHP treatment:** SW480 and SW620 cell lines were transfected with mirVana™ (Thermo Fisher Scientific) miR-92a-3p-mimic (Mimic), mimic negative control oligonucleotide (NC-mimic), miR-92a-3p-inhibitor (Inhibitor), inhibitor negative control (NC-inhibitor). Non-transfected cells were used as an additional control. Six hours after transfection, cells were exposed to increasing concentrations of 5-FU/L-OHP. After 24 hours of chemotherapeutics exposure, the viability of the cell lines was assessed by MTT assay. Viability percentage of SW480 (A) and SW620 (B) cells transfected with miR-92a-3p-mimic and control oligonucleotide was measured and contrasted with non-treated cells. Viability percentage of SW480 (C) and SW620 (D) cells transfected with miR-92a-3p-inhibitor and control oligonucleotide was also contrasted with non-treated cells. Comparison of inhibition rate curves for SW480 (E) and SW620 (F) cells transfected with miR-92a-3p-mimic, inhibitor, control oligonucleotides, and non-transfected cells. Statistical differences between responses to equal 5-FU/L-OHP concentrations were determined using ANOVA (A, B, C, and D), and EC50 shifts between experimental groups were assessed by non-linear regression (*p<0.05). Data represent the means ± SEM from n=3 independent experiments. IC50= inhibitory concentration of 5-FU / L-OHP required to decrease viability of each cell line by 50%. EC50= effective concentration of 5-FU / L-OHP required to decrease viability of each cell line by 50%.

**
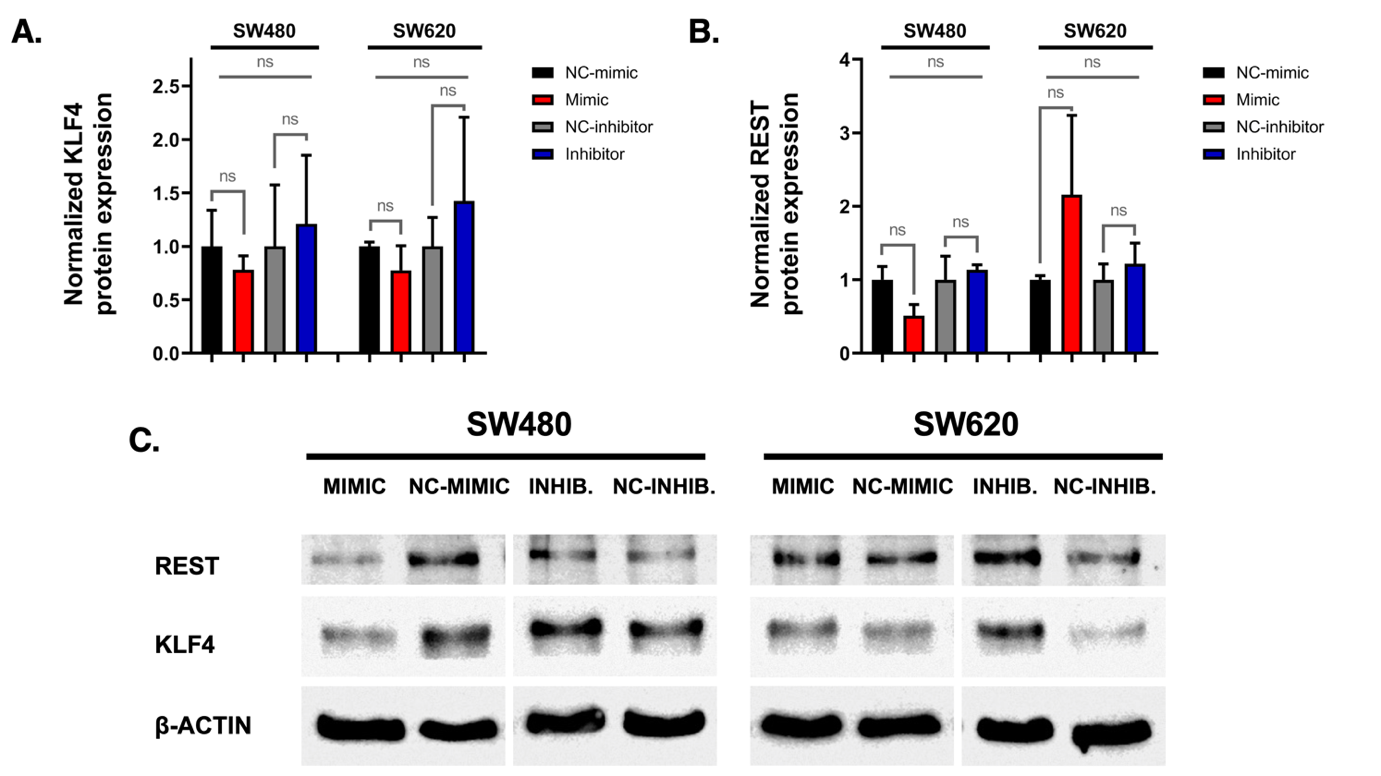
**

**Supplementary Figure S4. Protein expression of miR-92a-3p targets KLF4 and REST in SW480 and SW620 cell lines transfected with miR-92a-3p-mimic or inhibitors:** SW480 and SW620 cell lines were transfected with mirVana™ (Thermo Fisher Scientific) miR-92a-3p-mimic (Mimic), mimic negative control oligonucleotide (NC-mimic), miR-92a-3p-inhibitor (Inhibitor), inhibitor negative control (NC-inhibitor). proteins were extracted 24 hours after transfection for Western blot analysis. Western blot analysis showing the relative protein expression of (A) KLF4, and (B) REST upon miR-92a-3p mimic or inhibitor transfection in SW480 and SW620 cells. (C) Representative images from western blotting of KLF4, REST, and β-actin. Statistical differences in mRNA and protein levels were determined using Student’s T-Test or Kruskal-Wallis H test for two-group comparisons (square brackets) and ANOVA or Kruskal-Wallis H test for multiple group comparisons (straight superior lines) when corresponding (*p<0.05). Data represent the means ± SEM from n=3 independent experiments.
